# Intensive Family Intervention as Support for Professional Treatment: Evolution of Symptoms in a Diagnosed Case of Autism Spectrum Disorder

**DOI:** 10.3390/children9030400

**Published:** 2022-03-11

**Authors:** José María Salgado-Cacho, María del Pilar Moreno-Jiménez, María Luisa Ríos-Rodríguez

**Affiliations:** 1 Faculty of Psychology, University of Málaga, 29010 Málaga, Spain; mpilar@uma.es (M.d.P.M.-J.); mlurios@uma.es (M.L.R.-R.); 2 Hogar Abierto Foundation, 29001 Málaga, Spain

**Keywords:** autism, family support, case report, family intervention, ASD

## Abstract

This article shows the progress achieved in a child who has received professional treatment combined with a family intervention at home. It discusses a 22-month-old patient identified as showing warning signs of autism spectrum disorder (ASD), a diagnosis that was subsequently confirmed through a standardized ADOS-2 test at 31 months of age. To establish the initial working objectives, a functional diagnosis was carried out at 23 months of age using the Battelle Developmental Inventory; a maturational delay was detected, situating the child at an age equivalent to 16 months. A professional intervention was designed in an early childhood care center, complemented by family intervention, so that the hours in which the child participated in learning experiences were increased. Notable advances were made in the areas of cognitive and motor skills, with more standard scores than when initially evaluated. Progress was also observed (though to a lesser extent) in other developmental areas such as language total, adaptive behavior, and self-help, while slight delays in the areas of socio-emotional development and reasoning and academic skills were found.

## 1. Introduction

ASD is known as a pathology that manifests in some fundamental areas of neurodevelopment. The requirements for ascribing this disorder to a person were modified in the latest update of the Diagnostic and Statistical Manual of Mental Disorders-5 (DSM-5), with the three criteria defined in its previous version—significant impairments in social reciprocity and communicative intent, as well as restricted and repetitive behaviors—being reduced to only two criteria in the current diagnostic manual: deficits in communication and social interaction, and restricted and repetitive behaviors/interests [1]. The changed definition reflects the evolution towards a dimensional perspective of ASD as part of a continuum of neurodiversity rather than a disorder [2]. Over the years, this conceptual evolution has led to a broader range of symptoms in the diagnosis of autism [3]. However, this change in diagnostic criteria may also affect the prevalence of ASD [4], such as the increase in prevalence observed in recent decades that can partly be explained by increased professional awareness of the disorder, improved screening tools, and the broadening of diagnostic criteria [5]. The gradual increase could possibly be related to changes in the diagnostic criteria and case definitions, and increased recognition of ASD by professionals [6]. This allows earlier detection of warning signs and earlier initiation of interventions, both at the professional and family level.

The general recommendations for intervention in autism indicate that it is necessary to intervene early and intensively, even though this should not be attempted without the family since this option facilitates the achievement of treatment results. There is no “gold standard” in the treatment of ASD in Spain—at least none have been officially established—although the recommendations for choosing an intervention point out the importance of scientific evidence as a criterion of choice, although this should not be the only criterion [7]. The characteristics of the child, the skills of the family, the resources available, and the therapist’s own experience must also be considered.

Interventions implemented by parents at home facilitate the generalization and maintenance of the skills that have been acquired through professional treatment [8]. In fact, research suggests that even a low-intensity intervention implemented by parents can help to achieve this effect [9]. However, if the evolution of the severity of the symptoms of the affected person are taken into account, research to date shows different conclusions about the effect of family implementation [10]. This is not the case when the relationship between the number of hours of professional intervention and improvements in these symptoms has been studied, as a direct positive relationship has been found [11]. However, reality shows that it is likely that many families cannot afford to pay for an intensive treatment of at least 20 h per week.

If we focus on the studies that validate family intervention as a facilitating element of the symptomatologic evolution presented by the child, parental training in the core symptoms of autism, on the one hand, and strategies for intervention at home, on the other, have been identified as necessary [12]. Perhaps the level of knowledge and training acquired by parents to effectively implement intervention at home could be the aspect that explains the difference in the results offered by the scientific literature. Therefore, this acquired knowledge could be a moderator of the impact of family intervention on the evolution of symptoms in children with ASD [9]. If this question is validated, it should be taken into account when planning resources aimed at parental psychoeducation. This is especially relevant if we consider that family intervention is sometimes the only alternative when there is no possibility of implementing an intensive professional intervention.

For parents, the process that occurs from the beginning of feeling concern about the child’s development until an explanation of what is happening is provided can be a real odyssey [13], which can be complicated by new diagnostic classifications, a lack of knowledge of the health care system, and the needs of the child. Parents of children with ASD face severe stress, more so than with other developmental disorders, precisely because of the uncertainty of the diagnosis and prognosis [14]. Some research has confirmed that the support received by families after diagnosis is decisive in guaranteeing the implementation of the treatment to be provided to the children [15]. Precisely with the aim of accompanying parents through the initial grief period, the authors of this manuscript, together with other collaborators, have designed the PSICOTEA Program: family support for parents who receive a diagnosis of autism spectrum disorder in a child, which will be discussed in future publications.

Among the main elements describing the maternal perspective in caring for a child with ASD is emotional, family, and social overload [16]. There appears to be a relationship between the extent of childcare and the parents’ ability to effectively address their child’s behavior [17]. Parents of children with ASD have high levels of psychological stress, including parental stress, depressive symptoms, and social isolation [18]. This reactive psychopathology following the news of suspected or diagnosed ASD causes a decrease in parental caregiving abilities, paradoxically at a time when they will have to deal with numerous changes. As mentioned above, the interventions proposed for young children newly identified as being at risk for ASD are very demanding, requiring very active participation by the parents [19,20]. The relationship between parental sadness and the lack of compliance with therapeutic recommendations also seems to have been demonstrated [21].

Studies on family interactions when there is a child with ASD have focused mainly on the maternal–filial relationship, despite both parents being at home [22]. In fact, the most studied interaction skills have been maternal, exposing the long-term benefits of the mother–child relationship [23], and highlighting the emotional, family, and social overload of mothers involved in caring for a child with ASD [16]. However, it is becoming increasingly relevant to include both caregivers during treatment to ensure better adaptation to the child’s difficulties [24]. It should be taken into account that each parent will have a different relationship with the child, depending on the characteristics of the person, which will allow specific and complementary objectives to be worked on during the intervention.

On the other hand, improving parents’ knowledge levels about autism and teaching them strategies to manage their child’s problematic behaviors is known to decrease parental stress [25]. The benefits of involvement of the parents of children with ASD include an increase in the child’s communicative behavior, improved maternal knowledge of autism, and an improvement in the parent–child communication style, which also helps to reduce parental depression.

It should not be forgotten that children, whether they have developmental difficulties or not, spend a lot of time with their parents, and parents are the ones who know the child best, so the participation of parents in therapeutic interventions will provide an opportunity to better acquire and generalize the skills that are planned in the professional intervention.

For many authors, parental involvement is essential in any intervention with children who have autism spectrum disorder (ASD). This participation should be considered as an indispensable extension of the therapist’s intervention. However, for it to be effective, appropriate training, supervision, and follow-up are required, while ensuring that the parents have the appropriate competences and that strategies are applied correctly [26]. If these minimum implementation conditions are not met, family intervention may not be effective.

This study tries to understand the impact of a family intervention in a young child with ASD when this intervention is complementary to a professional treatment. To be effective, it is ensured that some minimum conditions are met during implementation, such as that the family has specific knowledge about the disorder and a professional accompaniment of the family is planned throughout the intervention.

## 2. Case Presentation

The subject of the case study is male and was 22 months old when his parents came to the pediatrics service to consult on the developmental abnormalities they had observed in the last 4 months. The patient was referred to the Early Childhood Care Unit, where a lack of eye contact, a lack of eye and auditory tracking, a lack of interaction, a lack of response to his name, a lack of language, a lack of symbolic play, and stereotypes such as tiptoe walking and flapping were identified, thus confirming the presence of ASD warning signs. Therefore, it was decided that he was suitable for intervention in an early childhood care center, a service which began that same week.

At the age of 30 months, he was recognized as having a disability of 39% (Class 3). (In Spain, disability is measured by degrees or percentages, which correspond to the impairment of the person. The five classes are classified as follows. Class 1: 0% disability; Class 2: between 1% and 24%, which is mild disability; Class 3: 25% to 49%, corresponding to moderate disability; Class 4: 50% to 74%, corresponding to severe disability; Class 5: more than 75%, corresponding to very severe disability.) At the age of 31 months, he was recognized as having Grade III dependency: strong dependency. (The assessment of the degree of dependency was performed by the administration and evaluated the degree of support necessary for the development of activities of daily living. There are three degrees according to severity: Degree I: moderate dependence; Degree II: severe dependence; Degree III: strong dependence.) The disability assessment was carried out by an interdisciplinary team from the autonomous administration, composed of a psychologist, a physician, and a social worker. The dependency assessment was carried out by a social worker from the local administration. The subject is currently 5 years old and is in the second year of kindergarten with Modality B schooling. (In Spain there are four schooling modalities, depending on the characteristics of the child. Modality A: ordinary group, full time; Modality B: ordinary group with attendance at the support classroom for variable periods; Modality C: ordinary center with a specific classroom; Modality D: special education centers.) Thus, he remains in a regular school with the support of teachers specializing in hearing and language, therapeutic pedagogy, and technical specialization in social integration. In the school center, he has also been assigned a personal assistant or “shadow teacher” in the classroom who accompanies him for 15 h a week to facilitate his adaptation to the school dynamics, a measure approved by the Delegation of Education of the Andalusian Regional Government through an agreement with the Autism Federation of the same community. Staff of the educational center suspected that he may have an associated comorbidity, attention deficit and hyperactivity disorder, due to his marked hyperactivity and lack of attention in class; however, the clinical judgment of the therapist involved in the case was that the child did not have enough symptomatology to justify evaluation for this disorder.

### 2.1. Case History

Peter (to facilitate the reading of the case, a pseudonym has been used to identify the patient) was born on 19 May 2015 via natural childbirth in which no epidural was used. He was born at term, only 1 day ahead of the due date. All pregnancy monitoring tests were performed without significant findings, except for the exudate test, in which a positive streptococcus result was detected in the mother. During delivery, two doses of antibiotics were applied, as foreseen in the health protocol, to prevent perinatal infection with this bacterium, approximately 30 min before delivery.

In the first months of life, most of the developmental milestones appeared normal (postural control, standing upright, and first steps, with no previous crawling). The most significant delay was that of the appearance of the first words and sentences.

At the time of detection, the mother was 39 years old. She has a degree in teaching and was working in a daycare center as an early childhood educator, although she is currently not in paid employment. The father was 38 years old and is a health psychologist; he was actively working at the time of detection but is currently in a private company. Peter has an older sister (aged 7 at the time of diagnosis) with typical development. There was no family history of ASD in either parent. Regarding the family history of central nervous system pathology, only one grandparent, the maternal grandmother, had been diagnosed with Alzheimer’s disease.

Until approximately 18 months of age, the parents reported that the child’s evolution was normal, without the presence of symptoms indicating a developmental anomaly. They described him as a happy, restless, and quite independent child. However, in retrospect, symptoms usually present in ASD, but which were perceived as normal at the time, were identified. The parents pointed out that the child showed a persistent liking for spinning objects and some stereotypical behaviors, such as hand flapping when he was happy (similar to his sister) and a tendency to tiptoe. Eye contact at the time of detection was low, and this deficiency was not so noticeable in the first year of life, with the parents assuring us that there was normalized eye contact at specific times, such as during breastfeeding.

### 2.2. Analysis and Description of Problem Behaviors

The Battelle Developmental Inventory was used for functional diagnosis. At the time of the evaluation, the case was 23 months old and had the following developmental ages in each of the areas evaluated: personal/social: 13 months; adaptive: 14; motor: 16; expressive communication: 9; receptive communication: 19; global communication: 12; cognitive: 22; total equivalent age: 16 months. On the basis of these results, an individualized treatment program was designed at the early childhood care center.

The child was evaluated by the neuropediatric service at 24 months of age. In this first assessment, a provisional diagnosis of “communication and language disorder” was established; however, 2 months later, the diagnosis was updated to “suspected ASD” by the professional at the early childhood care center after the intervention had begun. The diagnosis of ASD was confirmed at 31 months after application of the Autism Diagnostic Observation Schedule (ADOS-2) [27] with a score of 21. Scores between 8 and 11 points place the patient in the “autism spectrum” category and scores equal to or above 12 place the patient in the “autism” category, indicating that the child was strongly affected.

The child presented a large number of repetitive behaviors that were considered self-stimulatory. These behaviors were mainly visual, with a special preference for rotating objects or colored lights. Other self-stimulations with different sounds and textures were also observed. These self-stimulatory behaviors can interfere greatly in daily life and were, thus, a priority target in the design of the intervention. Other affected developmental areas included the absence of pointing skills, delayed oral expression, and inattention.

### 2.3. Establishment of Treatment Goals

According to the initial assessment, comprehensive work on the affected areas was established as a priority. Specifically, as stated in the first individualized treatment program carried out in August 2017, the normalization of motor (fine and gross), cognitive, communication, social, and language areas was established as a goal. Goal setting and evaluation was always carried out by the professional and was updated every 6 months on the basis of the objectives achieved with the combined intervention, prioritizing the work on the most affected areas. The professional referring the child was also in charge of the evaluation and training of the parents in terms of implementation of the intervention at home, thus ensuring that the family strategies were aligned with the objectives proposed in the biannual program. This document served as a guide for the joint professional–family work. Despite not being included in the initial document, it was also considered a priority to address the high number of self-stimulatory sensory behaviors since they decisively interfered with the usefulness of the work sessions. The individualized treatment program was updated every 6 months, adjusting it to the progress that had been achieved.

## 3. Treatment Planning and Design

Due to the openness of the professional in charge of the intervention with the child in the early childhood care center, and the training and experience of the father in the application of similar treatments, it was decided to carry out a joint planning procedure, attending to the therapeutic objectives derived through the professional assessment and others detected by the family. This procedure was considered appropriate because family-based behavioral interventions, in which family members participate as agents of change, are widely supported in the scientific literature [28,29,30].

The objectives and goals of the joint treatment followed the guidelines set out in the Individualized Treatment Program (ITP).

### 3.1. Implementation of the Treatment

The work organization had the format described below.

#### 3.1.1. Professional Treatment

Based on the clinical and diagnostic features of the case, an ITP was designed, corresponding to 12 early attention measurement units (EAMU) per month. Each EAMU corresponded to a 45-min work session and was distributed as follows: three weekly sessions of 45 min were established for the child, individually on Mondays and Thursdays, and in a group on Wednesdays.

For the intervention, a psychologist was assigned as case referent. A comprehensive intervention was applied on the basis of an evaluation of the symptoms that sought to impact all affected areas of development. The evaluation of deficits was carried out through the application of developmental scales, thus identifying the priority areas to work on with the child, which corresponded to the most affected areas.

The lead professional of the case, in accordance with the guidelines of the institution to which she belongs, considered applying strategies based on the Early Start Denver Model (ESDM) [31], with the aim of creating opportunities for communication between the child and the adult, allowing the child to learn new communication and social cognition skills.

The ESDM has shown promise as an effective practice for young children with ASD in improving outcomes, especially language and cognitive outcomes. However, in reference to the core symptoms of autism (social communication, adaptive behaviors, and repetitive behaviors) no significant progress has been observed [32]; therefore, it should be associated with additional treatments that compensate for this weakness.

For this reason, and as a complement to the chosen intervention model, several strategies of the Treatment an Education of Autistic and Communication Handicapped Children (TEACCH) methodology [33] and Applied Behavior Analysis (ABA) [34] were applied.

#### 3.1.2. Family Involvement in the Intervention

The family intervention followed the guidelines of the family-based intervention model, with the objective of complementing the treatment of the child with ASD through his family. This model incorporates the need for direct training and education of parents and close relatives, who carry out the intervention between the visits of the professional therapist. Family-based intervention in autism not only focuses on the needs of the affected person, but also pays attention to the family of the person with ASD, with the ultimate goal of empowerment and family autonomy. It is assumed that parents are the ones who can intervene more intensively during daily activities, which are an ideal context for learning [35].

For the correct implementation of the family intervention, quarterly meetings were planned between the therapist and the family at home to monitor the intervention, with the aim of evaluating and monitoring the objectives established in the ITP and correcting any errors that may have arisen during the implementation. To ensure that the parents were familiar with the basic elements of implementation, training was carried out in the following steps. Firstly, observation of the therapist’s intervention was facilitated through a Gessel camera in the early childhood care center; secondly, the intervention was carried out under direct observation in the same room as the parents; thirdly, sessions were held in which the parents intervened with the support of the therapist; finally, the intervention was carried out at home with the supervision of the therapist.

The structured family intervention was implemented by the father and had an average duration of between 4 and 6 h per week (individual work methodology, similar to that carried out at the early childhood care center in sessions of progressive duration of between 1 and 1.5 h). For the choice of the intervention methodology, we followed the premise of planning successful tasks related to new learning with progressive difficulty, avoiding a trial and error methodology. In order to favor the appearance of non-acquired behaviors and training in cognitive skills, highly motivating activities were designed, with strong social reinforcement and the use of sensory elements preferred by the child (such as the use of musical instruments when a correct answer was given) also used as reinforcement.

One of the main strategies identified by parents for the improvement of communication and language in the initial phase of treatment, who also considered it to be a potential precursor to reading, was the application of the ADRYNA method of speech learning with family support [36], a method pending empirical validation that facilitated the acquisition of a solid phonological awareness in this case. This method is an eminently phonetic intervention model that includes facial recognition, using clearly differentiated facial photographic models; it also reinforces associative memory and activates the motor commands of speech sensorially, with the help of facial molding. The intervention evolves in four phases: (1) differentiation, (2) repetition, (3) anticipation–generalization, and (4) knowledge communication strategies.

Regarding the progress achieved in the first year of intervention, Peter learned to read early. Although it was not initially a priority, this skill was acquired at 3 years of age, becoming a fundamental element for later learning, facilitating the structuring of tasks, and making it possible for the child to understand the instructions of tasks. Despite the early age at which this skill was acquired, it did not comply with a pattern of hyperlexia, appearing to be functional reading that helped to compensate for the difficulties with attention to oral language.

Table 1 shows the areas of initial intervention in the structured intervention carried out by the family, as well as the strategies used during the sessions.

An unstructured family intervention was also implemented by both parents, in which even the sister collaborated, following the indications. It was not possible to count the amount of dedicated time spent on this intervention, which was configured differently from education so that the family relations could be adapted to the learning needs of the child with ASD. This intervention consisted of small adaptations in the interactions with the child and in the layout of the home, favoring the stimulation of some less developed areas. Some of the strategies followed were:

- Inclusion of planned and permanent social reinforcement of the child’s eye contact with adults;
- Modification of the home layout so that the child was forced to make requests to obtain objects of preference that he could not reach and thus stimulate communication behaviors;
- Face-to-face placement with an adult at mealtime, favoring permanent interaction during a rewarding activity;
- Selective attention to well-formulated verbal requests to offer the requested object, and offering alternative behaviors to disruptive behaviors (as an example, a drum was offered when he was banging on the doors of a piece of furniture when he was looking for auditory self-stimulation).

A dietary intervention, where casein-containing products were eliminated due to a possible intolerance to cow’s milk protein at 26 months of age, was also initiated, and a normalized diet was resumed after 15 months.

## 4. Measurements and Tools

To evaluate the treatment’s effectiveness, three standardized developmental scales were used; they were always applied and evaluated by the same early childhood care center professional.

The first test used was the Spanish version of the Battelle Developmental Inventory [37]. This test consists of 341 items and includes specific scales to assess receptive and expressive communication skills, gross and fine motor skills, cognitive skills, and personal/social development. Measurements were taken with this instrument at 23, 34, and 57 months of age.

The second test used was the Spanish adaptation of the Merrill–Palmer Developmental Scale [38]. This scale has a cognitive battery and other subscales that measure gross motor skills, adaptive behavior and self-care, the socio-emotional area, and expressive language. Measurements were taken with this scale at 35 and 52 months of age.

The third test applied was the Autism Diagnostic Observation Schedule [27], which was used at 31 and 60 months of age. It is a standardized, semi-structured assessment of communication, social interaction and play, or imaginative use of materials for individuals suspected of having a diagnosis of autism. The ADOS-2 consists of a set of activities that allow the evaluator to observe whether or not certain behaviors that have been identified as important for the diagnosis of ASD at different developmental levels and chronological ages occur.

## 5. Results

### Evaluation of Treatment Effectiveness

**Figure 1:**
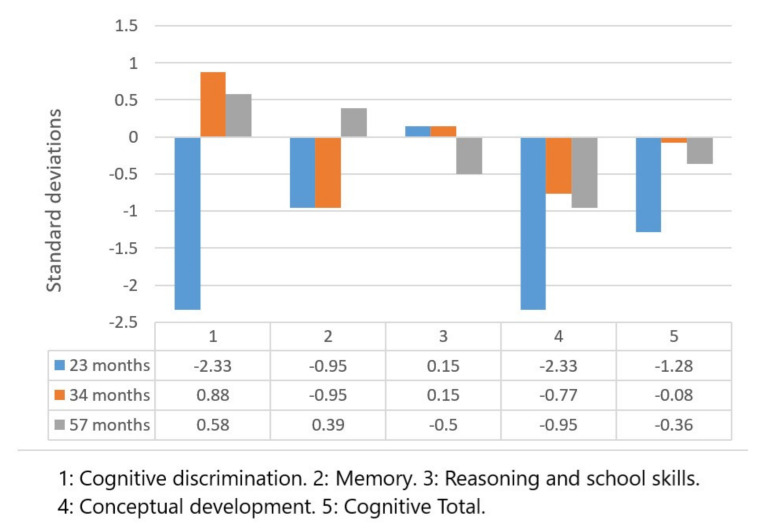
Cognitive scale scores on the Battelle Developmental Inventory.

**Figure 2:**
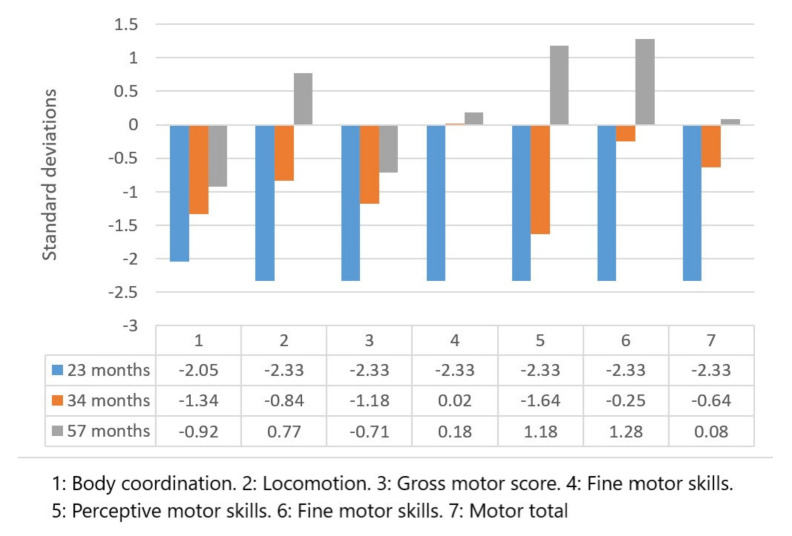
Motor scale scores of the Battelle Developmental Inventory.

The results of the Battelle Development Inventory are shown in Figure 1 and Figure 2, corresponding to the cognitive and motor scales, respectively. The scores are presented in terms of standard deviations (SD) above or below the mean of the population of his age, showing the level of development of the child in each of the areas evaluated in comparison with what would be expected at his chronological age.

The evolution seen for the different scales was as follows: Perceptual discrimination: total positive variation of 2.91 SD; Memory: total positive variation of 1.34 SD; Reasoning and scholastic skills: total negative variation of 0.65 SD; Conceptual development: total positive variance of 1.38 SD; Total for the cognitive area: total positive variation of 0.92 SD.

All the results obtained showed a positive evolution, except for reasoning and scholastic skills, in which a lower score than the initial score was obtained.

In the motor area of the same inventory, the following results were obtained for the evaluated areas: Body coordination: total positive variation total of 1.13 SD; Locomotion: total positive variation of 3.1 SD; Gross motor: total positive variation of 1.62 SD; Fine motor: total positive variation of 2.51 SD; Perceptual motor: total positive variation of 3.51 SD; Fine motor, with a total positive variation of 3.61 SD; Total motor area scores, with a total positive variance of 2.41 SD.

The results of the Battelle Development Inventory corresponded to the typical deviations of the cognitive and motor scales in the three chosen evaluative divisions.

**Figure 3:**
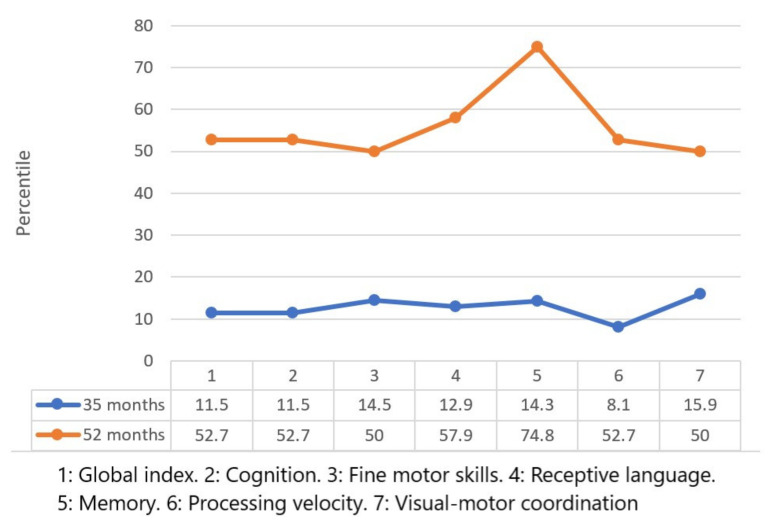
Scores of the cognitive battery of the Merrill–Palmer developmental scale.

**Figure 4:**
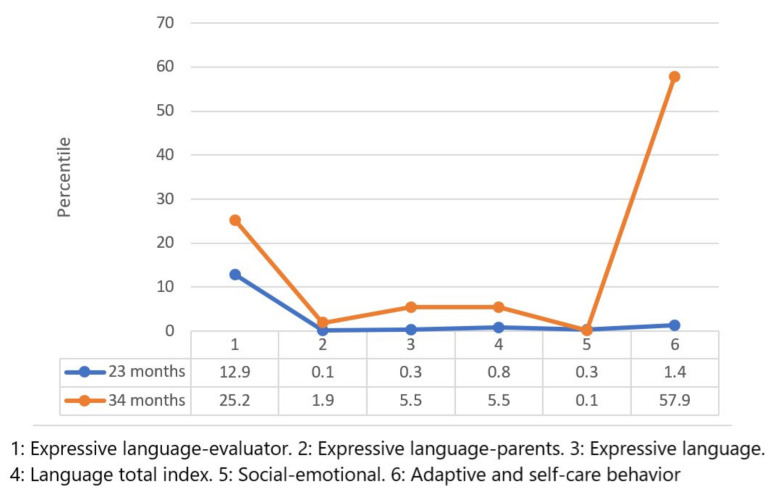
Scores for language areas, social–emotional and adaptive behavior, and self-care on the Merrill–Palmer Developmental Scale.

Figure 3 and Figure 4 show the results of the Merrill–Palmer Developmental Scale. The centile scores are shown, where the child’s position in each area is compared with a normative sample.

The following scores were found in the cognitive battery: Global index: positive development by 41.2 points; Cognition: positive evolution by 41.2 points; Fine motor skills: positive evolution by 35.5 points; Receptive language: positive evolution by 45 points; Memory: positive evolution by 60.5 points; Processing speed: positive evolution by 44.6 points; Visuomotor coordination: positive evolution by 34.1 points.

In the rest of the subscales, the following scores were found: Evaluative expressive language: positive evolution by 12.6 points; Parent expressive language: positive evolution by 1.8 points; Expressive language: positive evolution by 5.2 points; Total language index: positive evolution by 4.7 points; Socio-emotional: negative evolution by 0.2 points; Adaptive behavior and self-care: positive evolution by 56.3 points.

All the results obtained showed positive evolution, except in the socio-emotional area, where a lower score than the initial value was obtained. The degree of affectation in this area was the most marked and it was where the least evolution was found in the evaluations, which meant a change in priorities in the goals of the intervention as of the last evaluation.

The results obtained by the ADOS-2 scale identified the global score regarding the degree of behaviors associated with ASD that were present in the child. Module 1 was applied in the first evaluation at 31 months of age, and Module 2 was applied in the second evaluation at 60 months, with scores of 21 and 15 points, respectively. The implication of these results is presented in the discussion.

## 6. Discussion

There is no single scientific method to guide parents in raising a child with ASD. Likewise, there are no distinctive interventions that can meet all the needs of individuals with ASD [39]. The possible negative effects that a particular treatment may have on the family (19) must also be considered, such as the financial burden not covered by public health care or the fact that the child may spend less time with his or her family or peers.

Parents are the main contributors to their children’s adaptive functioning, and this is also true for children with ASD [40]. Involving parents in interventions for children with ASD helps to increase their knowledge about the diagnosis and treatment of this disorder, improving parent–child interactions and improving the choice of effective resources [41].

The importance of presenting data from case reports, as in this manuscript, has been considered for years as a means of advancing knowledge within the scientific literature [42] because it provides a rough version of the neuropsychological profile of a particular patient and how findings from other studies can be idiosyncratically applied to an individual. Case studies also help clarify how treatment design elements can have a treatment impact, despite the purity or modifications made to a treatment model.

In the case presented, an example of a coordinated intervention between professionals and family has been shown. The importance of applying this work methodology is based on the fact that the parents have the maximum knowledge of the child affected by ASD, which should be considered as an essential element for establishing the objectives of the professional treatment. Parental involvement helps parents to better detect the child’s needs and also increases their motivation to follow the treatment guidelines.

Significant progress has been observed in most of the areas evaluated, despite the fact that there was no linear progression. The scores obtained in the cognitive scale of the Battelle Development Inventory presented clear positive progression since the initial evaluation; accordingly, the best scores were obtained in the second evaluation at 34 months of age. In contrast, improvement could be observed in the scores obtained regarding the cognitive battery of the Merrill–Palmer Developmental Scale, where all scores in the first measurement were below the 20th percentile and reached average normative values in the second measurement.

The symptoms of ASD remain throughout life, and their severity is modulated by genetic predisposition and environmental factors, so early detection enables an intensive early intervention that can produce a better long-term prognosis [43]. In terms of the results obtained by the ADOS-2 test, a reduction in the total score obtained at the second evaluation was observed, but it must be considered that a different module was used, so the results are not comparable with each other. These results cannot be understood as a direct improvement in the symptoms presented by the child because the ADOS-2 only allows a calibrated comparison of severity between modules when the scores range between 1 and 10, and the patient’s score was not in this range. The total score obtained for each module shows the overall level of symptoms associated with autism in relation to other children with ASD of the same age and with the same language level as the patient. In Module 1, the cut-off points considered to be within the autism spectrum would be a score between 8 and 11 points, as scores considered to be within the autism category are from 12 points onwards. In this first evaluation, the child scored 9 points above the cut-off point for the autism category, indicating the significant severity of his symptoms. In the second evaluation, the module had a cut-off point for the autism spectrum category of 8, with the autism category having a score of 9 or higher. In this case, a score 6 points above this cut-off point was recorded. In spite of not being able to directly compare these scores, some interpretation of the results obtained with this test can be made, taking the limitations of not having research to validate these statements into account. On the one hand, the change of module made for the second evaluation assumed that the child had made a significant advance in language, evolving from the use of few or no words to a level of “language fluency”, which was a significant advance in itself [27]. On the other hand, if the cut-off points of each module are taken into account, this reduction in the total score could be understood as an improvement in the severity of the symptomatology presented by the child, if we consider the score obtained with respect to the scale with which each module is compared and not the total score between evaluations.

This study’s assessment, performed with the ADOS-2 scale, supported this theory of the permanence of the disorder despite advances in symptoms over time. According to the American Academy of Pediatrics, intervention should be initiated as soon as the warning signs are identified, but for an intervention to have an adequate impact, the National Research Council (2001) recommends a minimum intensity of 5 h per day for at least 5 days per week, which is hardly an acceptable requirement for most families, especially when a therapist must be in charge of delivering such an intervention. However, other studies have suggested the possibility of obtaining clinically significant results with fewer hours of direct therapist involvement when parental participation is active [44].

The intensity of an intervention, when extended to the family, is influenced by the degree to which parents implement appropriate strategies in the natural daily routines, and this element should be considered as a priority in the design of the intervention. On the one hand, it is necessary for parents to know the clinical or functional diagnosis of the child. On the other hand, it is also necessary for them to know the core symptoms associated with the disorder. Having this knowledge will make it easier for them to provide more immediate and contingent responses to the child’s difficulties [45]. This interaction can be understood as a continuum of behaviors ranging from more responsive to directive, with the responsive style being most associated with providing appropriate responses to not only the child’s communicative offers but also to other more subtle approaches, including gaze, physical orientation, facial expression, and gestures [46]. Interactions must also include a redirection of the child’s limited attention to the adult’s interests, with some aspects of a directive style being equally necessary in that interaction. Given that parents are the main modulators of family adaptation, when they are empowered to adequately understand the disorder and to attend to the needs of the affected child, it is easier to achieve better results in the evolution of the disorder [40].

Another aspect that is potentially associated with the progress found in the developmental areas evaluated was the early learning of reading. In children with normal development, the teaching of written language takes place when there is already an elaborate mastery of oral language at between 5 and 7 years of age in Spain, when it is assumed that the minimum prerequisites for this have been reached. However, it is not possible to establish a single age for the acquisition of this minimum level of maturity. According to Monfort and Juárez [47], written language serves as a support to speech because it helps to visualize the phonological structure of words, it provides a visual structure to fix oral language in the memory, it serves as a support to the acquisition of pragmatic skills because it can be used as a support to communication, and it makes the rules of the social use of language explicit. The teaching of reading can be one of the strengths of children with ASD because the high capacity for the association and matching of written words with images of represented objects tends to frequently facilitate the almost spontaneous learning of literacy [7]. Written representation, therefore, has several advantages in that it is a visual form of information that is permanent, observable for as long as needed, and physically manipulable.

Specifically, memory and selective attention to visual stimuli—to the detriment of auditory stimuli—were some of the strengths in the skills evaluated in the case, which facilitated the learning of reading. Its early acquisition also indicated the acquisition of an augmentative communication system that compensated for the limitations of attention and oral language comprehension, increasing the child’s communicative competence to understand instructions and other communication approaches by people in their immediate context.

In our case, significant development was observed in the cognitive and motor areas, with less development in the language and socio-emotional areas, areas which are more specifically associated with ASD. These results support the stability of the symptoms of the disorder throughout a person’s life, though they do not explain the low evolution of these symptoms over the studied months. The work priorities and strategies used with the case were mainly focused on the areas that experienced the greatest progress since it was considered at the beginning of the intervention that improvement in the cognitive area would create the basis for subsequent learning, whatever its nature. In the last evaluation carried out with the child, the intervention priorities were modified to focus on the work objectives of the less developed areas, such as language and interaction skills with other people; the results of this evaluation will be assessed in 12 months.

Although scientific evidence has mainly focused on the benefits of maternal involvement in the care of the child with ASD [48], it seems more advisable to include both parents in the family intervention. In this way, it would be possible to design strategies that could be implemented by each of them according to their strengths and weaknesses [49]. From this perspective, we would offer an optimized treatment that would include all the potential that the family can offer [50].

## 7. Conclusions

Parental involvement is essential in any intervention for children with ASD [51]. This involvement should be seen as an indispensable extension of the therapist’s intervention, but for it to be effective, appropriate training, supervision, and follow-up are required to ensure that parents have the right competencies and that strategies are applied correctly. This should involve a conceptual shift in which caregivers are considered equal members of the child’s professional treatment team, which means going beyond taking parents’ opinions into account and receiving their support, and getting parents actively involved in training sessions and clinical decision making to identify the goals and opportunities for children, as well as to generalize and maintain recently acquired skills for themselves and other family members [39]. However, it should also be considered that high levels of parental stress explain the low involvement in interventions with children, so the emotional support of caregivers should also be included as a goal of the intervention [40,52]. Therefore, it should be kept in mind that family intervention may not be effective in all cases, particularly if minimum implementation conditions are not met. If no models, practice, or feedback are provided, parents may not correctly apply the strategies in addition to not feeling confident in their skills at home, which may reduce the number of actions they can potentially apply [26]. Our proposal is that early childhood care centers should be responsible for providing these psychoeducation services to parents, and they should accompany families in the search for emotional stability as a protective factor against parental stress, as these are fundamental elements required to achieve progress in interventions carried out with children.

In this case, in addition to their previous academic training—as a psychologist and a teacher—the parents received specific training from the professional in charge of the child’s treatment, first through observation, then through supervised practice and subsequent monitoring of the implementation being carried out.

This study had an important limitation that makes it difficult to generalize it to the treatment of other children with ASD-associated symptomatology: the training and profession of the parents of the patient favored the design and application of the treatment. Therefore, it is crucial that future work continues to investigate what types of parental education and training are necessary to facilitate adequate family involvement in the treatment of children with these characteristics.

Further research is needed to identify the specific elements that make an intervention effective. In turn, it is important to have effective assessment tools that can establish the type of treatment that best suits the characteristics of the patients, the agents who should be involved in the intervention (therapists, parents, teachers, or a combination of these) or the minimum intensity at which an adequate impact will be achieved by the intervention. It will also be necessary for the design of the intervention to consider other factors that may influence its success, such as the patient’s age, the degree of disability developed, or the need for intervention by other complementary support services.

## Figures and Tables

**Table 1:** Affected or planned areas as preventive work objectives and strategies designed for the intervention.

Identified Need or Objective to Prevent	Strategy
Excessive liking for colored lights (visual self-stimulation)	Overexposure strategies with the following elements: - A rotating colored light bulb; - A light table with different color options; - A fringe lantern with colored lights.
Excessive liking for noises when hitting furniture (sonorous self-stimulation)	Overexposure strategies and establishment of a place for this reinforcement: - Creation of a “noise corner” with sound elements produced by vibration or when banged, such as drums, maracas, and rattles.
Excessive liking for rotating elements (visual self-stimulation)	Overexposure strategies: - Daily use of different rotating toys on a workbench.
Excessive liking for jumping and other stimuli that activate the vestibular system	Strategies for overexposure and establishment of a place for this reinforcement: - Placement of a trampoline in the family home, freely accessible by the child; - Regular use of elastic swings, solid swings, and see-saws.
Speech stimulation and prevention of texture hypersensitivity in the mouth.	- Mouth stimulation with toothbrushes with different textures; - Mouth stimulation with an electric toothbrush; - Mouth stimulation with a vibrator/face massager.
Absence of the ability to indicate	- Use of a glove with a magnet on the index finger to catch objects with that finger; - Reinforcement with colored lights when touching a requested object with the index finger; - Use of the index finger with a phoneme primer game, supported with high social reinforcement.
Very marked delay in speech	- Reinforcement of words and approximations of words emitted in requests; - Placement of toys and preferred items in high places, forcing help-request behavior.
Prevention of sensory hypersensitivity to touch	- Massages all over the body with different elements, including hands, massagers of different textures, electric massagers, and vibrating blankets; - Manipulative games with different textured elements.
Prevention of sensory hyperreactivity (noise)	- Use of various musical instruments in the sessions, initially for listening to and then for playing, increasing tolerance to the increased volume.
Prevention of sensory hyperreactivity (smells)	- Use of games with different smells elements that were initially pleasant; later, strong smells that he had to identify were introduced.
Stimulation of cognitive skills	- Completing series, memory cards, matching identical figures, and associating animals with the sound they make, among others.

## Data Availability

The data presented in this document were collected in the evaluation reports carried out by a professional external to the investigation and are guarded by the main investigator.
